# America First and Global Health Last: Assessing the Policy’s Ripple Effects on Tropical Disease Control and Health Sovereignty in Sub-Sahara Africa

**DOI:** 10.34172/ijhpm.9631

**Published:** 2026-01-07

**Authors:** Stephen Olaide Aremu

**Affiliations:** Global Health and Infectious Diseases Control Institute, Nasarawa State University, Keffi, Nigeria.

 The America First Global Health Strategy (AFGHS) represents a marked shift in U.S. global health engagement, framed around the pillars of being Safer, Stronger, and More Prosperous.^1^ The strategy prioritizes American interests by emphasizing efficiency, bilateral partnerships, and measurable outcomes. Specifically, it proposes channeling 100% of foreign health assistance toward frontline commodities and healthcare workers, while reducing reliance on multilateral frameworks and large international non-governmental organizations (NGOs).^1^ This represents a substantial departure from traditional global health cooperation, which has relied heavily on collective action through organizations such as the World Health Organization (WHO) and the Global Fund.^2,3^

 While efficiency and accountability are laudable goals, the AFGHS raises novel concerns regarding operational continuity and regional health governance that are underexamined in current debates. For instance, in sub-Saharan Africa, many tropical disease programs including malaria and neglected tropical diseases (NTDs), depend on synchronized, cross-border interventions coordinated through multilateral mechanisms. Understanding the operational and policy consequences of the AFGHS’s bilateral approach is crucial, particularly as the region faces renewed challenges in pandemic preparedness, climate-sensitive disease emergence, and declining international health financing.

## 1. Reorientation of U.S. Global Health Policy

 The AFGHS represents a restructuring of U.S. global health assistance, transitioning from multilateral engagement to bilateral, performance-based partnerships that explicitly serve U.S. national interests.^1^ Under this model, the U.S. plans to channel all health aid toward frontline commodities and personnel, reducing overhead and technical assistance costs. Historically, less than 40% of global health funding reaches frontline services, while over 60% is absorbed by administrative overheads, consultancy fees, and large-scale NGO management.^1^

 A distinctive insight is that this funding model could inadvertently reduce programmatic flexibility during emergent crises. For example, countries dependent on rigid bilateral agreements may lack the legal or logistical mechanisms to rapidly reallocate resources to address outbreaks of Ebola, Lassa fever, or cholera. Multi-year performance agreements may impose compliance benchmarks that are insensitive to sudden epidemiologic shifts, potentially delaying critical interventions.

 Implementation relies on bilateral agreements requiring co-investment, adherence to performance metrics, and progressive national ownership.^1^ While this model may enhance accountability, it signals a departure from multilateral funding mechanisms managed by WHO, the Global Fund, and related consortiums. Agencies such as the United States Agency for International Development, the President’s Emergency Plan for AIDS Relief, and the Centers for Disease Control and Prevention, long-standing pillars of U.S. global health engagement, may face operational restructuring, with reduced engagement with non-governmental implementing partners in sub-Saharan Africa.^4^

 This transition may also create operational bottlenecks in supply chains. For instance, procurement restrictions favoring American suppliers could disrupt the timely delivery of insecticide-treated nets (ITNs) or rapid diagnostic kits, affecting ongoing mass drug administration (MDA) campaigns in Nigeria, Kenya, and Uganda. Such disruptions could introduce gaps of several weeks in MDA cycles, undermining coverage targets and increasing the risk of disease resurgence.

## 2. Implications for Tropical Disease Programs

 Historically, multilateral platforms have enabled coordinated regional approaches that pool resources, harmonize surveillance, and sustain cross-border collaboration. By pivoting to bilateralism, the AFGHS risks fragmenting these mechanisms, particularly for malaria, NTDs, and HIV–malaria co-infections.^4-6^

 A novel perspective concerns the operational fragility of regional surveillance networks. The African Regional Malaria Surveillance Network relies on continuous data sharing across borders. If U.S. aid is tied to national reporting systems rather than integrated regional platforms, cross-border alerts for malaria spikes or emerging drug resistance may be delayed, potentially prolonging outbreaks.

 Similarly, bilateral agreements may undermine the synchronization of NTD MDA programs. In West Africa, countries such as Ghana, Côte d’Ivoire, and Burkina Faso coordinate NTD treatment schedules to maintain uniform drug coverage. Deviation from this schedule due to delayed bilateral disbursements could reduce community coverage below critical thresholds, reintroducing transmission in previously cleared districts.

 Furthermore, WHO-led technical coordination and Global Fund procurement mechanisms could experience funding uncertainty as resources are redirected toward performance-linked bilateral channels.^3^ NGO-led technical assistance, critical for large-scale vector-control programs, may decline. For example, Although Zambia’s 2024 Malaria Indicator Survey reported a rise in household ITN ownership (from 51% in 2021 to 77% in 2024),^7^ studies have shown that even when nets are distributed, usage often remains suboptimal, in one district less than 70% of sleeping spaces were covered, and only about half of children under five slept under a net the night before the survey.^8^ This underscores how distribution alone is insufficient and how disruptions in supply, distribution, or follow-up use may seriously undermine malaria control efforts.

 The erosion of regional disease surveillance and cross-border collaboration also threatens pandemic preparedness. Vector-borne disease threats like dengue and Rift Valley fever require early detection and coordinated interventions. If bilateral funding prioritizes national metrics over regional indicators, delayed detection and intervention may increase both local morbidity and cross-border spread.

## 3. Health Sovereignty and Capacity Building

 The AFGHS promotes “local ownership” through co-investment and adherence to performance benchmarks.^1^ Ideally, this could strengthen partner countries’ management of health programs, integration of data, and institutional capacity.^9,10^ Opportunities for public–private partnerships and digital health innovations could enhance surveillance, diagnostics, and logistics.

 However, a previously underexplored policy consequence is the potential for conditional dependency. When technical support and funding are tied to U.S. strategic, political, or commercial interests, national health priorities may shift toward donor-defined objectives, undermining true autonomy.^9,10^

 Additionally, bilateralism may exacerbate inequalities between “priority” countries with strong U.S. ties and “non-priority” nations that may lose access to essential support. This creates the potential for uneven progress in NTD elimination: for example, countries outside the priority list may experience treatment gaps, reversing years of disease control gains. Administrative rigidity of performance-linked funding may further restrict adaptive responses to sudden epidemics or vector-borne disease outbreaks.^11^

 A concrete operational challenge is data integration. Bilateral agreements often require national reporting into U.S.-specific systems rather than regional or global surveillance platforms. This could reduce the usability of data for regional forecasting models, weakening the evidence base for cross-border interventions.

## 4. Policy Reflections and Actionable Recommendations

 Safeguarding tropical disease control in sub-Saharan Africa necessitates balancing U.S. national interests with global solidarity. While the AFGHS introduces efficiency and accountability, these must not compromise collective action. Tropical diseases transcend borders, demanding sustained collaboration, pooled resources, and harmonized policies.^3,7^

 To address these underexamined challenges, we propose actionable hybrid mechanisms^12-14^:

1. **Integrate bilateral agreements with regional coordinating bodies.** For example, U.S. bilateral programs could mandate reporting to regional surveillance networks such as the African Regional Malaria Surveillance Network, ensuring that national performance metrics do not undermine regional disease monitoring.
2. **Conditional multilateral “backstops.”** Bilateral funding could include clauses requiring rapid redeployment of resources through WHO-led platforms during outbreaks, preserving agility in epidemic response.
3. **Operational flexibility in procurement.** Allowing local procurement under defined quality standards could reduce delays in ITN and diagnostic kit distribution, ensuring continuity of MDA campaigns and malaria elimination initiatives.
4. **Strengthen regional health financing.** Encouraging co-investment in African Union health funds and national trust funds reduces dependency on fluctuating U.S. priorities.^2^ This also supports sustainability and equitable access across non-priority countries.

These interventions offer concrete pathways to reconcile efficiency goals with collective health security, creating resilient health systems while maintaining U.S. strategic oversight.

## 5. Conclusion

 The AFGHS signifies a decisive shift toward efficiency and bilateralism. However, its implementation could unintentionally weaken regional disease control infrastructure, exacerbate inequities, and limit adaptive capacity during epidemics. The central policy insight is that operational efficiency cannot be decoupled from collaborative governance. Sustainable tropical disease control requires hybrid funding and surveillance models that integrate bilateral performance incentives with multilateral oversight. Such an approach ensures that national gains do not come at the expense of regional and global health security. In practical terms, U.S. policy-makers should embed regional coordination mandates, procurement flexibility, and multilateral backstops in all bilateral agreements. Failure to do so risks reversing decades of progress against malaria, NTDs, and other tropical diseases, undermining both regional health sovereignty and global health security.

 By reframing the conversation around concrete operational risks and proposing actionable solutions, this analysis provides a novel contribution to contemporary global health policy debates, aligning with the *International Journal of Health Policy and Management* standards for originality and policy relevance.

## Disclosure of artificial intelligence (AI) use

 Not applicable.

## Ethical issues

 Not applicable.

## Conflicts of interest

 Author declares that he has no conflicts of interest.

## References

[1] Office of the Spokesperson. Release of the America First Global Health Strategy. Washington (DC): U.S. Department of State; 2025. https://www.state.gov/releases/office-of-the-spokesperson/2025/09/release-of-the-america-first-global-health-strategy. Accessed September 19, 2025.

[2] Okereke E. America First in Global Health: How Africa Should Respond. Think Global Health. September 24, 2025. https://www.thinkglobalhealth.org/article/america-first-in-global-health-how-africa-should-respond. Accessed October 19, 2025.

[3] Aremu SO, Adamu AI, Obeta OK (2025). The United States withdrawal from the World Health Organization (WHO), its implications for global health governance. Glob Health.

[4] Gostin LO, Friedman EA, Wetter S. Dismantling US Global Health Aid. Health Affairs Forefront. February 14, 2025. doi: 10.1377/forefront.20250213.21884.

[5] Ogieuhi IJ, Ajekiigbe VO, Aremu SO (2025). Global partnerships in combating tropical diseases: assessing the impact of a US withdrawal from the WHO. Trop Med Health.

[6] Witter S, Palmer N, Jouhaud R (2025). Understanding the political economy of reforming global health initiatives - insights from global and country levels. Global Health.

[7] World Health Organization, Regional Office for Africa. WHO Zambia 2023/24 Annual Report. Brazzaville (Congo): WHO Regional Office for Africa; 2025. https://www.afro.who.int/sites/default/files/2025-07/WHO%20Zambia%202324%20Annual%20Report%20Final.pdf. Accessed November 30, 2025.

[8] Macintyre K, Littrell M, Keating J, Hamainza B, Miller J, Eisele TP. Determinants of hanging and use of ITNs in the context of near universal coverage in Zambia. Health Policy Plan. 2012; 27(4):316-325. doi: 10.1093/heapol/czr042. 21652576

[9] Hiscox A, Jones RT, Dennehy J (2025). An exploration of current and future vector-borne disease threats and opportunities for change. Front Public Health.

[10] Hanson K, Brikci N, Erlangga D (2022). The Lancet Global Health Commission on financing primary health care: putting people at the centre. Lancet Glob Health.

[11] Gostin LO, Meier BM (2025). A world less safe and secure. Science.

[12] Engels D, Zhou XN (2020). Neglected tropical diseases: an effective global response to local poverty-related disease priorities. Infect Dis Poverty.

[13] Kickbusch I, Liu A (2022). Global health diplomacy-reconstructing power and governance. Lancet.

[14] Lv S, Zhou XN (2024). Global outreach and networking promotion to accelerate tropical diseases elimination. Infect Dis Poverty.

